# A model for predicting utilization of mHealth interventions in low-resource settings: case of maternal and newborn care in Kenya

**DOI:** 10.1186/s12911-018-0649-z

**Published:** 2018-07-17

**Authors:** Stephen Mburu, Robert Oboko

**Affiliations:** School of Computing and Informatics, University of Nairobi, P.O. Box 30197-00100, Nairobi, Kenya

**Keywords:** Behaviour science, Design science, Fit, mHealth, Predictive modeling, Self-efficacy, Short message service (SMS), Structural equation modeling, Utilization

## Abstract

**Background:**

In low-resource settings, there are numerous socioeconomic challenges such as poverty, inadequate facilities, shortage of skilled health workers, illiteracy and cultural barriers that contribute to high maternal and newborn deaths. To address these challenges, there are several mHealth projects particularly in Sub-Sahara Africa seeking to exploit opportunities provided by over 90% rate of mobile penetration. However, most of these interventions have failed to justify their value proposition to inspire utilization in low-resource settings.

**Methods:**

This study proposes a theoretical model named Technology, Individual, Process-Fit (TIPFit) suitable for user-centred evaluation of intervention designs to predict utilization of mHealth products in low-resource settings. To investigate the predictive power of TIPFit model, we operationalized its latent constructs into variables used to predict utilization of an mHealth prototype called *mamacare*. The study employed single-group repeated measures quasi-experiment in which a random sample of 79 antenatal and postnatal patients were recruited from a rural hospital. During the study conducted between May and October 2014, the treatment involved sending and receiving SMS alerts on vital signs, appointments, safe delivery, danger signs, nutrition, preventive care and adherence to medication.

**Results:**

Measurements taken during the study were cleaned and coded for analysis using statistical models like Partial Least Squares (PLS), Repeated Measures Analysis of Variance (RM-ANOVA), and Bonferroni tests. After analyzing 73 pretest responses, the model predicted 80.2% fit, and 63.9% likelihood of utilization. However, results obtained from initial post-test taken after three months demonstrated 69.1% fit, and utilization of 50.5%. The variation between prediction and the actual outcome necessitated improvement of mamacare based on feedback obtained from users. Three months later, we conducted the second post-test that recorded further drop in fit from 69.1 to 60.3% but utilization marginally improved from 50.5 to 53.7%.

**Conclusions:**

Despite variations between the pretest and post-test outcomes, the study demonstrates that predictive approach to user-centred design offers greater flexibility in aligning design attributes of an mHealth intervention to fulfill user needs and expectations. These findings provide a unique contribution for decision makers because it is possible to prioritize investments among competing digital health projects.

**Electronic supplementary material:**

The online version of this article (10.1186/s12911-018-0649-z) contains supplementary material, which is available to authorized users.

## Background

To exploit opportunities provided by mobile penetration in developing countries, there is proliferation of technology innovations aimed at improving healthcare service delivery [1–4]. This is the motivation behind numerous mobile health (mHealth) interventions aimed at overcoming challenges like poor infrastructure, staff shortages, and limited budgets that characterize low-resource settings [5–7]. Despite these initiatives, a global observatory survey conducted by World Health Organization (WHO) and International Telecommunication Union (ITU) revealed that majority of mHealth systems are weak platforms that have failed to transit to actual practice [8]. Prior studies have also attributed failure of mHealth interventions to misalignment to realistic needs and expectations of the target users [9–11]. Since most mHealth initiatives in Sub-Sahara Africa are donor-funded projects, we argue that low utilization of most of these interventions may be due to poor understanding of users, tasks and technology context during design. Several case studies have revealed that design of some of mHealth systems is based on “perceived problems”, then “pushed” for adoption and use by consumers who were least involved in designing the intervention [8, 12].

**Fig. 1:**
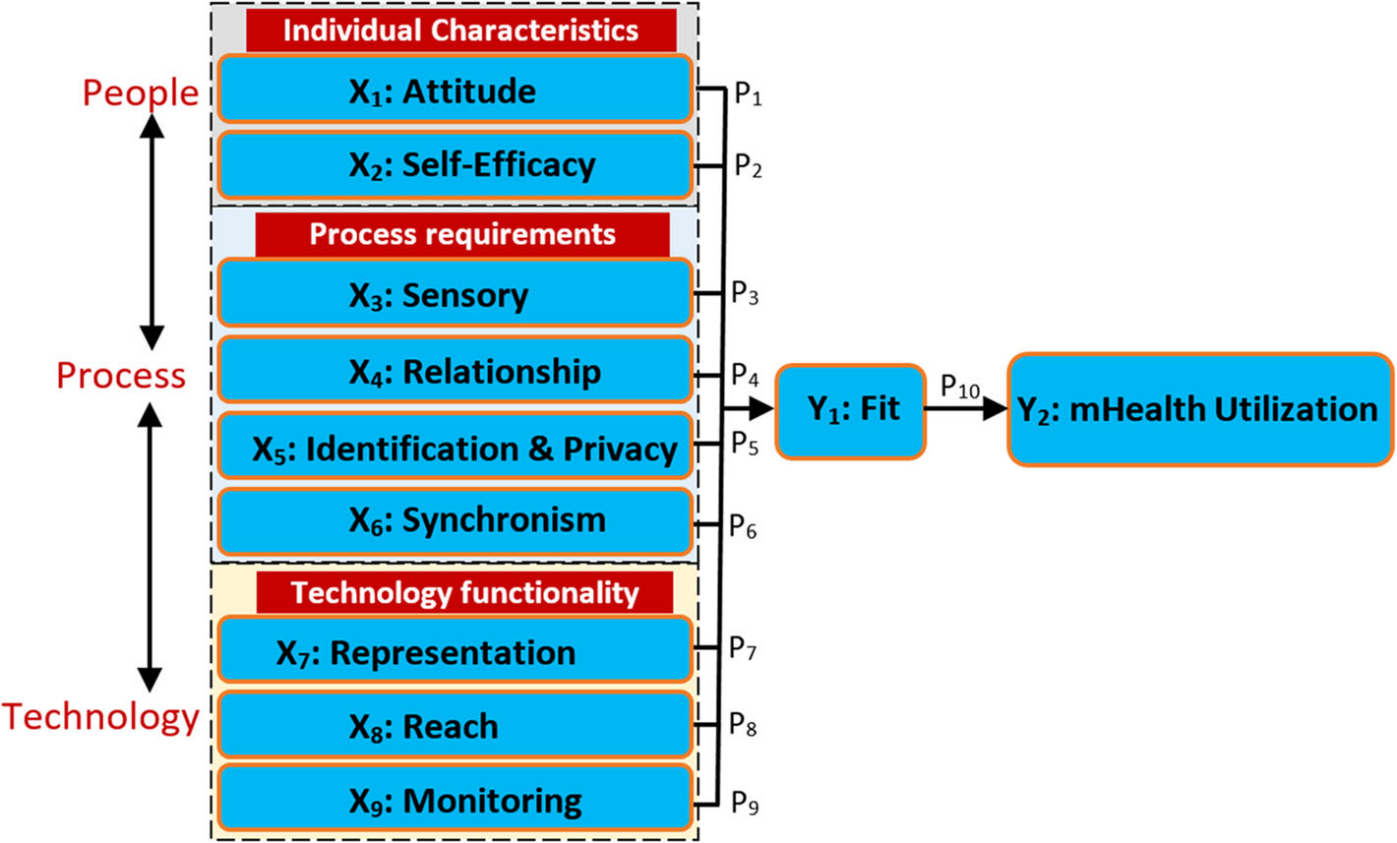
TIPFit model consisting of predictor variables (X_1_-X_9_) hypothesized to influence fit. Consequently, fit determines utilization of mHealth interventions as depicted by P_10_

To scale up utilization of mHealth innovations, there is need for user-centred evaluation of design specifications to predict usage behaviour after workplace implementation. Some of the reviewed studies on technology adoption have demonstrated how to predict utilization based on theoretical knowledge of causal connections [13–15]. For example, Davis and Venkatesh [14] used Technology Acceptance Model (TAM) to predict acceptance and use of a new system based on perceived usefulness. The same approach was used by Bhattacherjee and Premkumar [15] to provide empirical evidence on predictive approach to user acceptance testing. This study therefore builds on similar approaches to predicting acceptance and use of mHealth interventions in low-resource settings. Due to gaps identified in the reviewed models and theories [16–22], we derived a structural model for predicting utilization of mHealth interventions. The model called *TIPFit* comprises of predictor variables X_1_ to X_9_ shown in Fig. 1; hypothesized to influence *fit* and utilization of an mHealth intervention. **TIPFit** is an acronym derived from **individual, process**, **technology,** and **fit** constructs. Similar to studies by Strong et al. [21] and Davis and Konsynski [22], **fit** is configured as a surrogate measure of user acceptance to determine temporal changes toward usage of mHealth artifacts. Justification and detailed reasoning regarding inclusion of each construct as a predictor variable is provided in the methods section.

To validate the model, we conducted within-subjects repeated measures quasi-experiment. The validation process was done in a practical scenario to investigate how well user’s perceptions predicted utilization of *mamacare* prototype. Mamacare is an integrated mobile and web-based application optimized to run on low-cost smartphones because most health facilities in low-resource settings have limited access to computers, power and broadband internet. Furthermore, WHO [10] recommends use of mobile phones to facilitate timely delivery and access to healthcare services. There is no doubt that acceptance and use of mHealth innovations has the potential to achieve Sustainable Development Goals (SDGs) aimed at reversing maternal and newborn deaths by 2030 [23].

## Methods

To build a strong case for the predictive method employed in this study, we first justify inclusion of eleven TIPFit variables classified into five constructs: individual, process, technology, fit and utilization of mHealth [14, 15, 23, 24].

### Attitude (X_1_)

Prior studies in behaviour science have shown that attitude influences one’s judgment on certain behaviour, subject or action [25–27]. Therefore, inclusion of attitude as a predictor variable was informed by our pre-study experience, and empirical findings from studies that are based on Theory of Planned Behaviour (TPB) [16, 26, 27]. In TIPFit model, attitude is crucial in measuring patients’ and caregivers’ perception before and after exposure to an intervention. We hypothesized that attitude changes over time as benefits of an intervention becomes more realistic due to continued use.

### Self-efficacy (X_2_)

Self-efficacy as a predictor variable was derived from Technology Acceptance Model (TAM) and Computer Self-Efficacy (CSE) [17, 20]. The variable is intended to measure one’s ability to use technology to access healthcare services and information. In particular, we used this predictor to measure one’s ability to use mobile phones to access maternal care services and information in rural areas.

### Sensory requirements (X_3_)

Sensory requirements as a predictor variable was derived from Process Virtualization Theory and Impact of IT (PVT-IT) [18, 22]. Overby and Konsynski [22, 28] demonstrated that sensory requirements of touch, smell, sight and taste makes it difficult to virtualize some physical processes. Moreover, Overby [18] argues that if a process requires sensory experience of smell, taste or touch, it would be more difficult to replicate these senses in a virtual (electronic) environment. For example, during routine maternal care visits, clinicians use medical devices to physically take clinical tests such as temperature, blood pressure, blood sugar, and haemoglobin. Although some of these vital signs may be taken remotely using wireless sensors, it may be difficult or costly to deploy such technologies in low-resource settings. This is why sensory requirements variable is crucial in predicting the degree to which mobile phones and point-of-care devices can be used to fulfil sensory requirements in maternal and newborn care.

### Relationship (X_4_)

Relationship as a predictor variable was derived from PVT-IT [18, 22] to investigate the degree of interaction between caregivers and patients in remote areas. We observe that in clinical processes, relationship is important because it builds mutual trust between patients and caregivers.

During physical encounter, verbal and non-verbal communications convey vital information resulting to mutual trust and better inter-personal relationships [28]. Although multimedia technology may be used to simulate such interaction, limitations of cost and infrastructure in low-resource settings make multimedia-based interventions unsustainable.

### Identification and privacy (X_5_)

Identification refers to proof of one’s identity while privacy refers to confidentiality of health information. This variable derived from PVT-IT [18, 22] was largely informed by our pre-study experience during focus group discussions. We noted that prove of identity in clinical processes like diagnosis is essential if patients and caregivers are to share sensitive information. For example, a HIV-positive patient may be reluctant to receive reminders on adherence to antiretroviral (ARV) regimen through mobile phones. On the other hand, clinicians may be reluctant to perform diagnosis and prescription electronically to avoid compromising patient’s privacy [1, 29].

### Synchronism (X_6_)

Synchronism as a predictor variable was derived from PVT-IT to measure degree to which time-critical processes are completed with minimal delay [18, 22, 28]. In medical practice, synchronism is crucial in emergency cases like preeclampsia that require urgent clinical attention. Our pre-study experience revealed that delays in detecting complications related to pregnancy and postpartum are some of the major causes of deaths in developing countries [4, 5, 13]. Therefore, synchronization was included as a predictor variable to measure degree to which use of mobile phones and point-of-care devices reduce delays in executing clinical tasks.

### Representation (X_7_)

This variable was derived from PVT-IT [18, 22] and Task Technology-Fit (TTF) [19, 21, 22] to investigate capabilities of technology to simulate or present information relevant to clinical processes [18, 22]. For example in telemedicine, mobile phones may be integrated with wireless sensors and multimedia tools to provide remote consultation between patients and doctors. However, due to poor connectivity, it becomes difficult to provide such services in low-resource settings [28]. In this study, we used representation as a predictor variable to measure degree to which mHealth artifacts could be used to simulate a clinical process like diagnosis.

### Reach (X_8_)

Inclusion of reach as a predictor variable was informed by empirical findings relating to PVT-IT [18, 22]. The variable is a measure of technology capability to provide sufficient access to maternal care services at reduced cost and time. In reviewed studies, it is evident that most mHealth interventions fail to provide adequate access to maternal care services and information due to long distances, inadequate health facilities, and cultural barriers [5–7, 28]. Therefore, we used reach to investigate how mobile phones and point-of-care devices could provide sufficient reach by reducing time and cost of accessing maternal care services and information.

### Monitoring (X_9_)

This variable was adapted from PVT-IT [18] to measure capability of technology to monitor patient’s health status. During antenatal and postnatal care, mothers are required to make at least four visits to monitor their progress. However, in remote areas, most patients fail to honour such visits hence resulting to complications like stillbirth and haemorrhage. To provide sufficient patient monitoring in such places, mobile-based interventions that use wireless body sensors may be considered. Nevertheless, such interventions may not be feasible due to limitations relating to poor infrastructure, cost, privacy and cultural beliefs. In this study, we used the variable to predict degree to which mobile devices could be used to provide sufficient patient follow-up in low-resource settings.

### Fit (Y_1_)

In the context of this study, fit refers to perceived usefulness, suitability or benefits of a planned intervention. Justification of including fit as a mediating variable was informed by studies conducted by Goodhue and Thompson [19], Strong et al. [21], and Overby and Konsynski [22]. Our reasoning is that perception on fit in terms of user, task and technology requirements determine utilization of an mHealth intervention [14, 17–19]. We posit that the higher the perception on fit, the higher the likelihood of utilizing an intervention.

### mHealth utilization (Y_2_)

In this context, utilization is the behaviour of using technology to accomplish some tasks [19]. Justification of including utilization as the outcome (dependent) variable was based on the premise that intention to use or usage of an mHealth system or product is influenced by perceived fit [14, 15, 19, 21]. In this study, we used the variable to measure the intention or utilization level of an mHealth intervention [30–32].

#### TIPFit constructs as predictor variables

The ability to make predictions from a structural model depends on knowledge of causal relationship between predictor variables and the outcome [24]. Therefore, to test the predictive power of TIPFit model, we converted the causal relationships depicted using P_1_ to P_10_ into Structural Equation Model (SEM). The structural model comprises of a system of multi-linear regressions represented using the following equation:

$$ {\mathbf{Y}}_{\mathbf{j}}={\boldsymbol{\upbeta}}_{\mathbf{i}}{\mathbf{X}}_{\mathbf{i}}+{\boldsymbol{\upvarepsilon}}_{\mathbf{i}} $$

In the equation, X_i_ represents the predictor variables (X_1_, X_2_…X_9_) hypothesized to influence fit [33–35]. The ***Y***_***j***_ term denotes two variables, i.e., Y_1_ and Y_2_ that represents fit and utilization of mHealth respectively. The term ***β***_***i***_ (***β***_1_ to ***β***_9_) represent path coefficients P_1_ to P_9_ used to determine the effect of each variable on fit. Path P_10_ on TIPFit is an aggregate coefficient used to measure cumulative effect of fit on mHealth utilization. The error term, i.e., Ɛ_i_ represents unexplained variations in each of the predictor variable X_1_ to X_9_.

To measure the degree to which a variable predicts changes in fit and utilization, we operationalized the model into ten hypotheses. Table 1 lists a set of null hypotheses denoted by H_0_1 to H_0_10 used to test the causal relationships represented by paths P_1_ to P_10_ on TIPFit model. Inferences from the hypotheses were drawn from path weights (***β***_***i***_) computed using Partial Least Squares (PLS) algorithm in SmartPLS [36].

**Table 1:** Hypotheses for predicting fit and utilization of mHealth

Path	Prediction hypotheses
H_0_1	Attitude has no significant change on fit before, and after use of mHealth intervention
H_0_2	Self-efficacy in use of mobile devices has no significant change on fit before, and after mHealth intervention
H_0_3	Sensory requirements have no significant change on fit before, and after use of mHealth intervention
H_0_4	Relationship requirement has no significant change on fit before, and after use of mHealth intervention
H_0_5	Identification and privacy requirements have no significant change on fit before, and after use of mHealth intervention
H_0_6	Synchronism requirement has no significant change on fit before, and after use of mHealth intervention
H_0_7	Representation capability of technology has no significant effect on fit before, and after use of mHealth intervention
H_0_8	Reach capability of technology has no significant change on fit before, and after use of mHealth intervention
H_0_9	Monitoring capability of technology has no significant change on fit before, and after use of mHealth intervention
H_0_10	Perceived fit has no significant change before, and after use of mHealth intervention

Source: Researchers’ TIPFit hypothetical model

#### Operationalizing TIPFit into structural path model

**Fig. 2:**
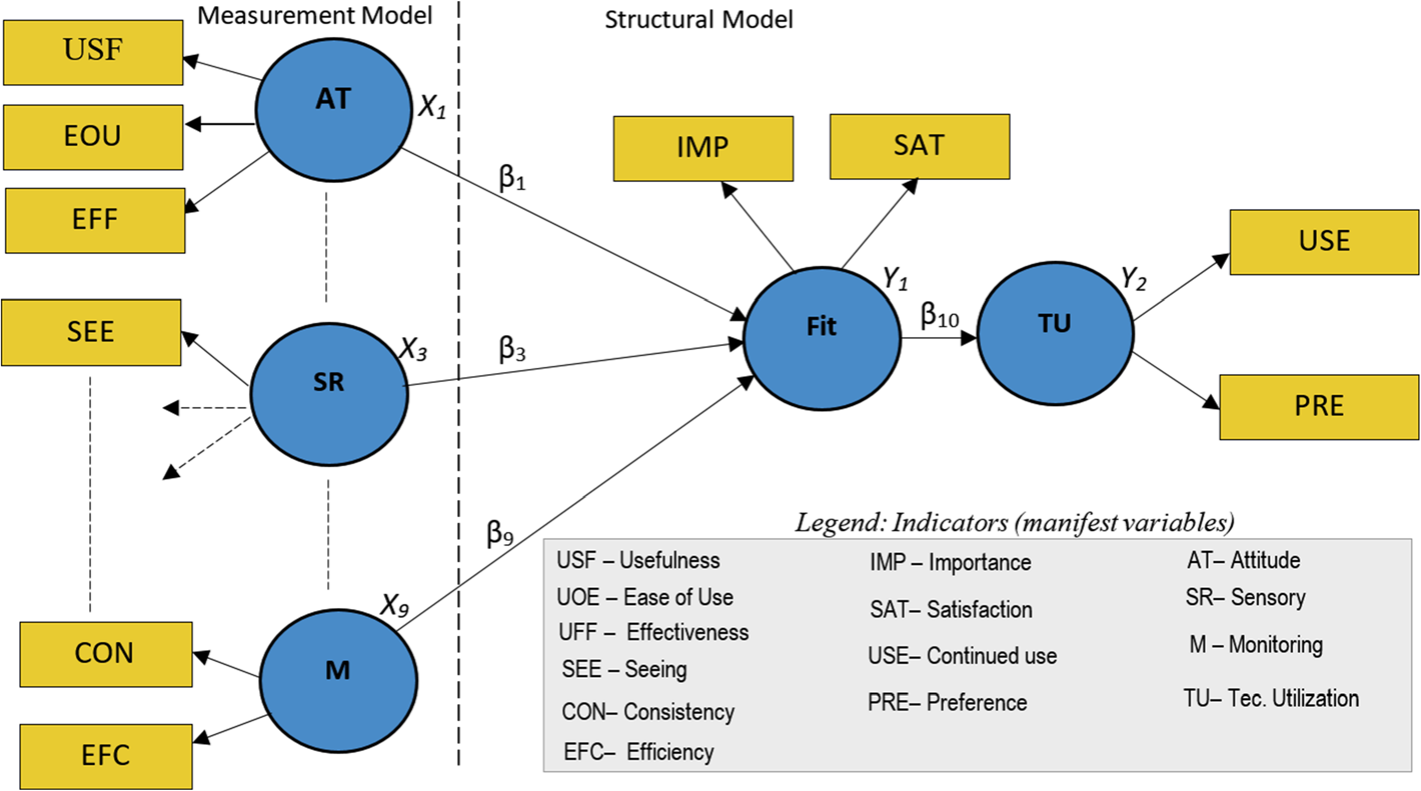
Operationalizing TIPFit into a graphical path model for analysis using path modeling software tools like SmartPLS

To test hypothesized cause-and-effect relationships, we operationalized TIPFit into a path model consisting of two parts namely measurement, and structural model. Figure 2 shows how three of the nine variables were operationalized into measurement, and structural models.

The measurement model represents predictor variables (X_1_ to X_9_) measured using manifest variables represented using initials in the leftmost boxes. The manifest variables shown on the legend of the diagram are scale items in the measurement instruments provided as Additional files 1, 2, 3, 4 and 5. The inner part of the model comprises of path coefficients from β_1_ to β_9_ hypothesized to influence fit. Consequently, β_10_ is used as a measure of how fit as an intervening variable functionally determines utilization of an mHealth artifact. It is this graphical model that formed the basis for predicting fit and utilization of an intervention using SmartPLS 2.0.

### Study design

The study was conducted for a period of six months starting from 5th May to 31st October 2014. This was after we obtained ethical approval issued by the Kenyatta Hospital/University of Nairobi Ethics Research Committee (KNH/UoN-ERC) on 23rd November 2013. Our study setting was the Maternal and Newborn Healthcare (MNH) section of a rural hospital called *Kimbimbi Sub-county Hospital*. The hospital, located in Kirinyaga County 110 km from Nairobi serves patients; most of whom are farmers from Mwea Rice Irrigation and Settlement Scheme.

#### Maternal care intervention

**Fig. 3:**
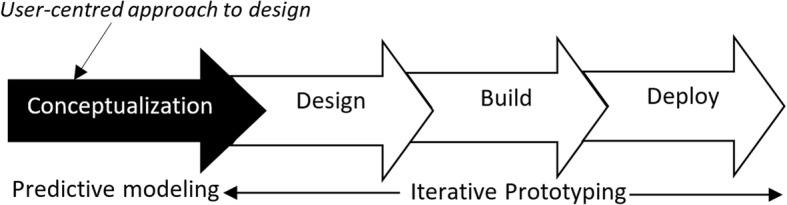
Approach employed in the development of mamacare prototype that was used to support maternal and newborn care

To develop mamacare, we employed user-centred design to understand the study environment, user needs, and maternal care process. Figure 3 shows the approach used; a customized model of agile development methodology.

During conceptualization phase, TIPFit was instrumental in measuring perceived fit of mobile-based intervention as a basis for predicting post-deployment utilization [14, 15]. Some of the user-centred techniques employed to understand the target users and clinical tasks in MNH include storyboards, mock-ups, interviews and focus group discussions. Feedback obtained from these interactions was used as the basis for the next phase of designing *mamacare*; a mobile and web-based prototype. Mamacare is an acronym derived from two words, i.e., mama that stands for “mother” across many languages, and care referring to maternal and newborn healthcare.

**Fig. 4:**
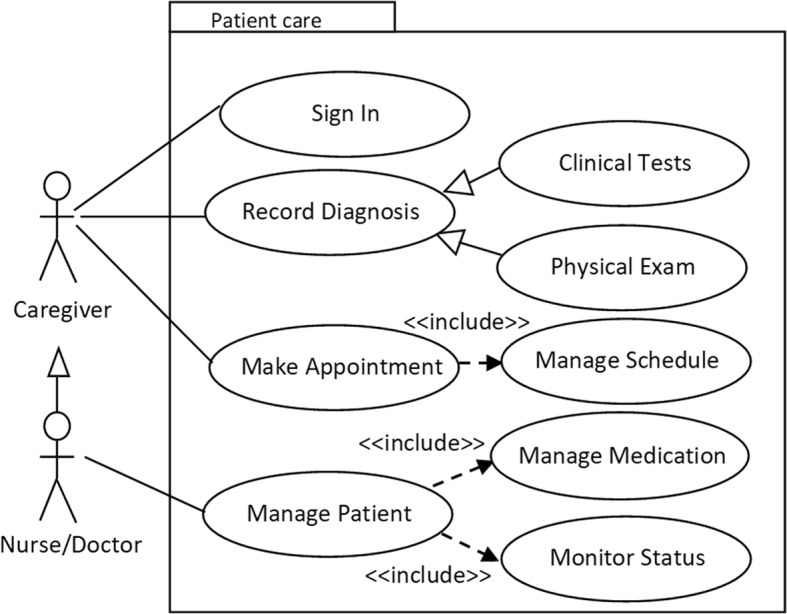
Use case model depicting the interaction between mamacare system and caregivers

In design phase, we used unified modeling language (UML) tools to align the planned intervention to user requirements identified during conceptualization. Figure 4 is a sample use case diagram that depicts interaction between mamacare and clinicians (caregivers) that were involved in the study.

**Fig. 5:**
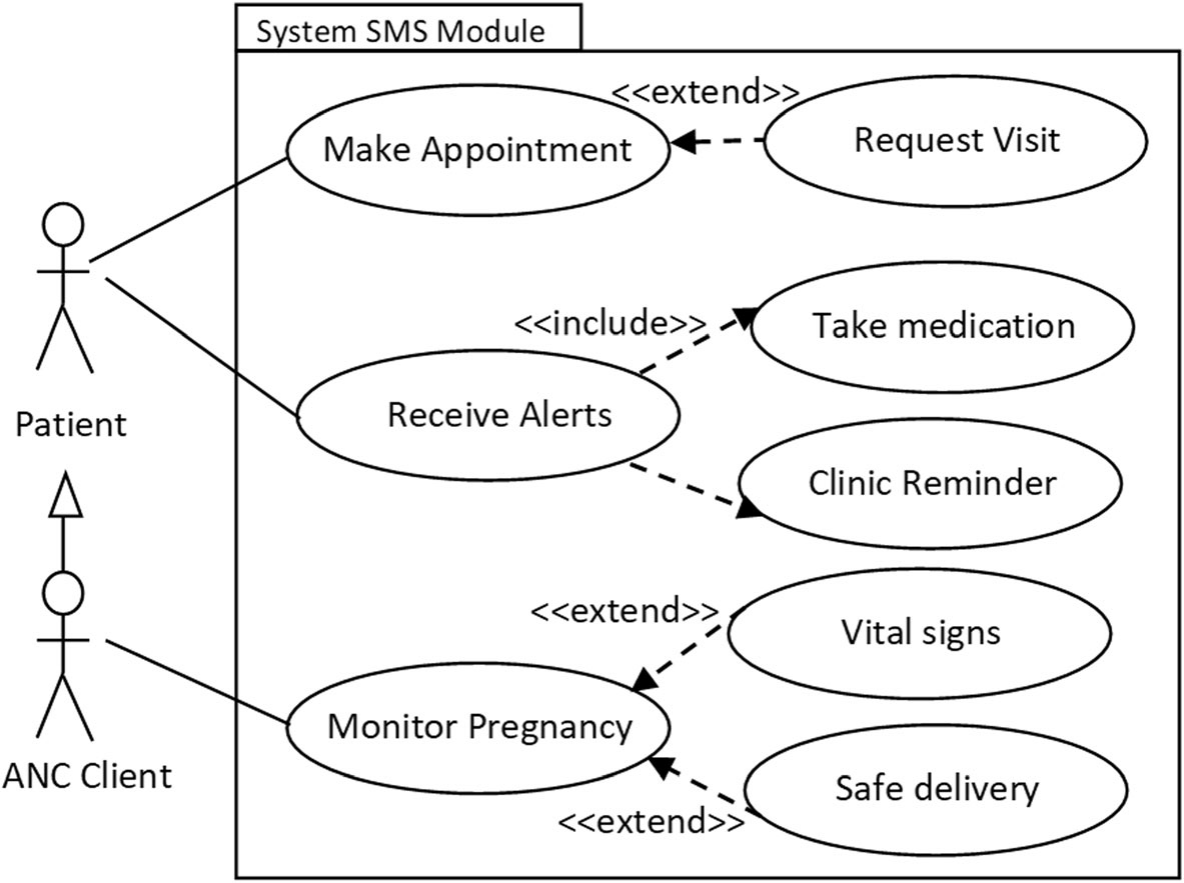
Use case model depicting the interaction between mamacare system and registered patients

To improve access to maternal care services and information through mobile, Fig. 5 shows a sample use case diagram depicting interaction between mamacare and patients.

**Fig. 6:**
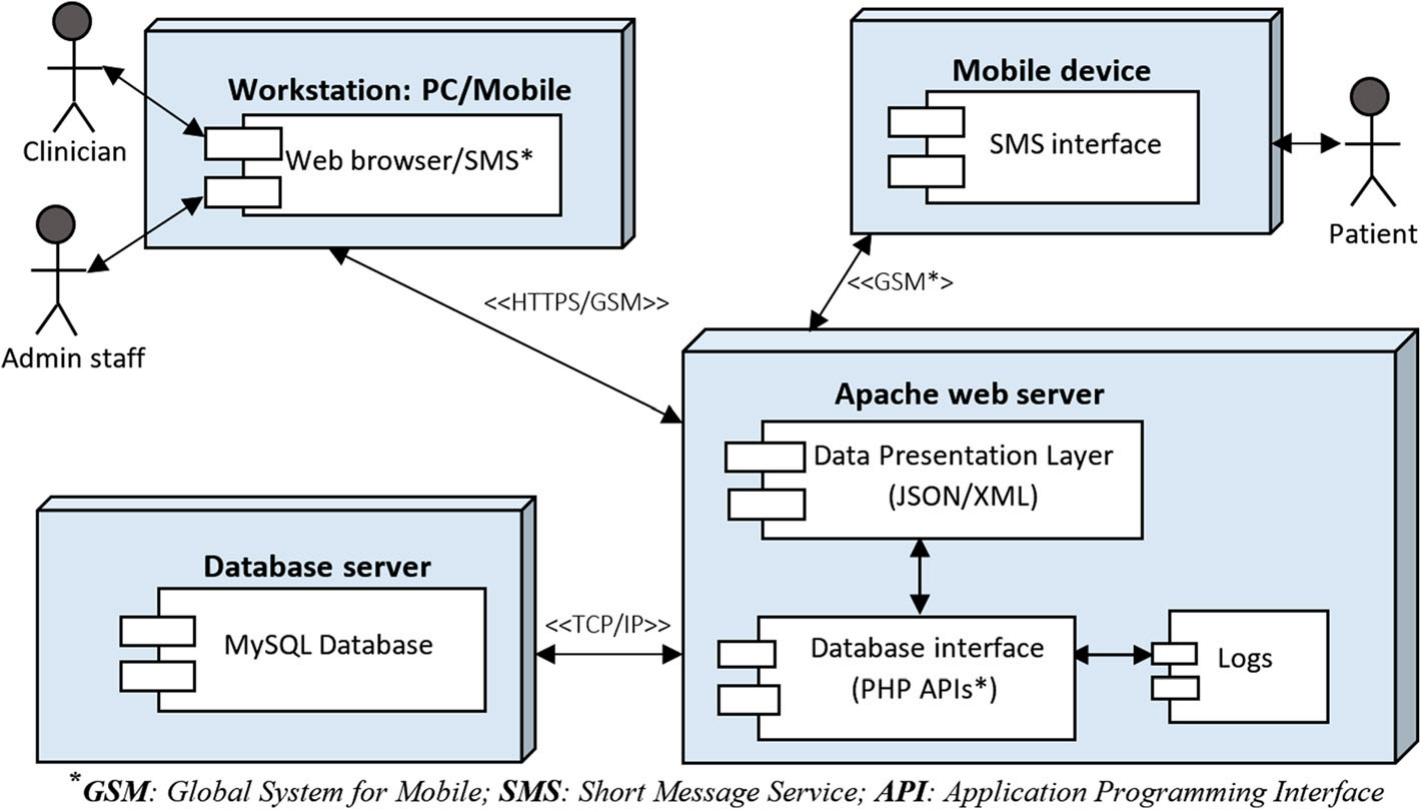
Mamacare deployment architecture. The clinicians and admin staff have controlled access to integrated web and mobile interface; while patients can only receive or send SMS messages via their own mobile phones

During the build phase, we used web development tools like HTML5, CSS3 and JavaScript to implement the web portal used by caregivers to process and manage patients’ health records. The Short Message Service (SMS) module was implemented using open source SMS Server Tools3 while the back-end was implemented using MySQL, Apache web server and PHP. Figure 6 depicts the architecture used to deploy mamacare in the study setting. The primary database server was installed in the hospital while a backup server was hosted at University of Nairobi for redundancy and security purpose.

**Fig. 7:**
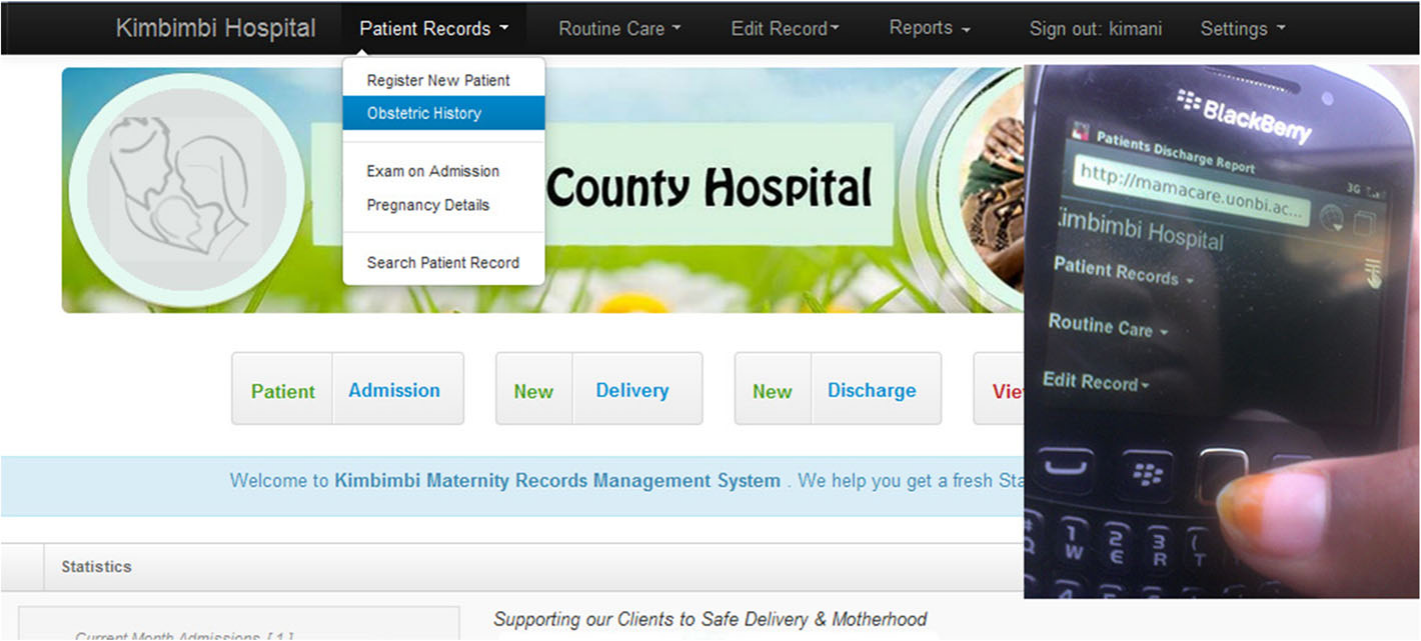
Mamacare web portal on computer on the left; and mobile phone interface inset on the right. **a** *Vital signs SMS.* **b** *SMS-based TCA reminder (Esther is a pseudonym)*

To enhance user experience, the web interface was designed to adapt to multiple device profiles depending on the screen size and orientation. Figure 7 shows how the same web portal appears on desktop computer and mobile phone. This responsive behaviour makes mamacare suitable for use in places with limited access to computers.

**Fig. 8:**
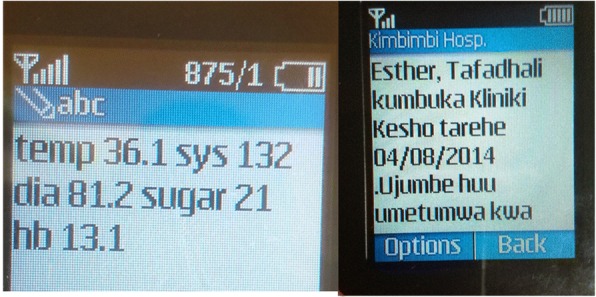
**a** The screen image on the left shows vital signs sent as SMS message to mamacare backend system. **b** on the right shows a sample SMS reminder generated based on maternal profile; and sent to a pseudonym (Esther) that represents an actual patient receiving mamacare services

Before mamacare was deployed, we agreed with the hospital management that the system was complementary to standard procedure for managing antenatal and postnatal patients. The complementary mechanism involved sending SMS messages on appointments, danger signs, safe delivery, nutrition and preventive care to registered patients. Mamacare also receives vital signs for temperature, blood pressure, and blood sugar to enhance monitoring of mothers and their children. Figure 8(a) shows vital signs received via SMS while Fig. 8(b) shows a sample SMS reminder on clinic appointment otherwise referred to as “To Come Again (TCA)” in maternal care context.

#### Design of repeated measures quasi-experiment

**Fig. 9:**
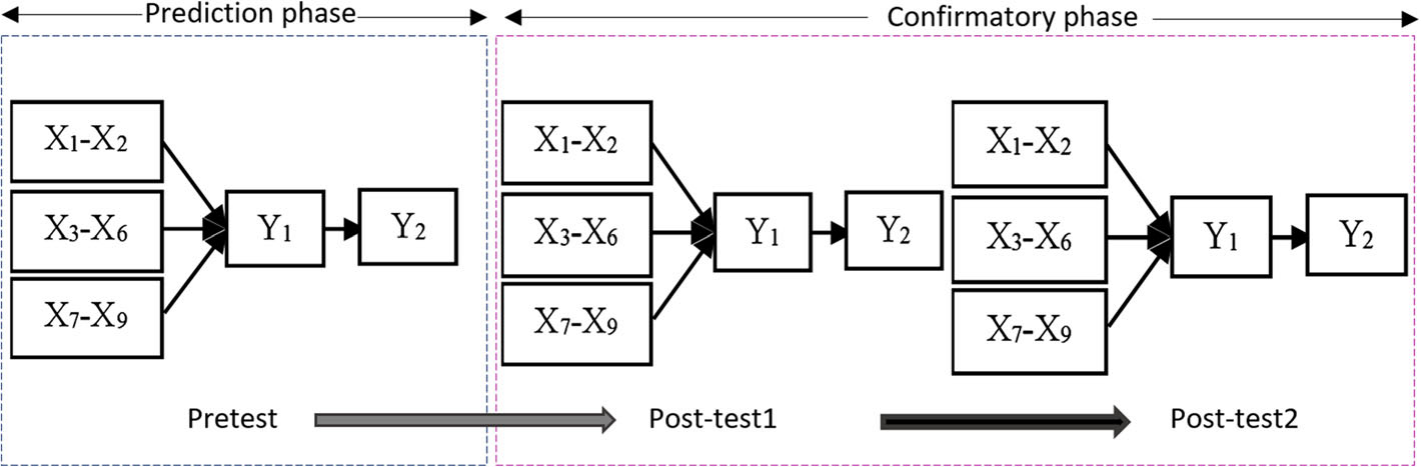
Repeated measures quasi-experiment to predict fi and utilization of mHealth interventions in low-resource settings

To measure the predictive power of TIPFit model, we used quasi-experiment to repeatedly measure responses from the same group of respondents before, and after intervention. Despite shortcomings of quasi-experiments in terms of internal and external validity, single-group repeated measures design is desirable in clinical environment where randomization may raise political, ethical or legal issues. In this regard, our study protocol approved by KNH/UoN-ERC required use of study designs that would not deny subjects benefits of the planned intervention. This was the main reason that influenced choice of single-group (within-subjects) repeated measures design. In this design, each subject served as her own experimental control hence making it possible to detect the effect of predictor variables on fit and utilization of mamacare. Figure 9 shows how the three measures were taken before, and after exposure to mamacare intervention for a period of six months.

Before the intervention, a pretest (T_0_) was used to measure perceptions based on benefits of mamacare communicated to participants during health education sessions organized by the hospital. Three months after the subjects were exposed to intervention, we conducted the first post-test (T_1_) to compare with predicted outcome. To compare the initial post-test outcome with reactions after prolonged use, we conducted the second post-test (T_2_) using the same tools employed in the initial post-test evaluation.

#### Sampling and inclusion of study population

During the inception stage of this study, we visited the Maternal and Newborn Healthcare (MNH) section of Kimbimbi Sub-county hospital to review the antenatal and postnatal registers. The reviewed registers had a total of 226 women most of whom were receiving either antenatal or postnatal care services. To get a representative proportion from this population, we used simple random sampling with age, education, gestation, residence, and ownership of mobile phone as inclusion criteria. Empirical findings from related studies have shown that factors like age, environment, and education influence individual’s attitude and ability to use technology [16, 17, 26, 27]. The gestation factor was considered because during pregnancy, women tend to change their attitude and ability to perform tasks. The ownership of mobile phone was also important because the purpose of the present study was to investigate utilization of mobile devices in maternal and newborn care. Therefore, to get an optimal sample from the population of 226 registered patients, we used the following formula to determine the optimal sample size:

$$ n=\frac{{\mathrm{z}}^2\mathrm{x}\;\mathrm{p}\;\mathrm{x}\;\mathrm{q}\;\mathrm{x}\;\mathrm{N}}{{\mathrm{e}}^2\left(\mathrm{N}\hbox{-} 1\right)+{\mathrm{z}}^2\mathrm{x}\;\mathrm{p}\;\mathrm{x}\;\mathrm{q}} $$

In the equation, *n* represents the sample size; *z* = critical value at 5% significance level; *p* = sample proportion (degree of variability) set as conservative value of 50%; *N* is size of finite population; *e* is the level of precision set at ±5%; and *q* = 1 – p. By taking *N* = 226; z = ±1.96 based on 5% significance level; p as 50% (0.5); e = 0.05; and *q* = 0.5 (1–0.5) we obtained our sample size as follows:

$$ n=\frac{1.96^2\times 0.5\times 0.5\times 226}{0.05^2\left(226-1\right)+{1.96}^2\times 0.5\times 0.5}=143 $$

This implies that a sample of at least 143 subjects was required for the study. After contacting these subjects through mobile calls and SMS, only 95 women accepted to attend a formal training session organized through the hospital. During the two-hour training, benefits and limitations of using mobile phones were communicated to the participants. Based on this information, 79 participants were recruited after they agreed to participate in the study by signing consent forms. The other 16 participants refused to participate due to issues relating to financial constraints, attitude and privacy.

Although the number of participants recruited was half of the expected, it was sufficient to get reliable inferences. Goodhue et al. [37] demonstrated that a sample of 40 subjects is sufficient to achieve reliable results in PLS. Furthermore, Overby and Konsynski demonstrated that a sample of 60 subjects is sufficient to detect small and medium effect [22, 38, 39].

#### Measurements

The study used three measures at different points in time to investigate the predictive power of a hypothetical model. The measurement instruments used before and after intervention were based on indicators derived from TIPFit model.

Before mamacare was deployed, we conducted a pretest as a baseline for predicting post-deployment utilization based on perceived benefits. The measurement instruments included basic demographic scale items such as age, education and gestation assumed to influence attitude and ability to use technology. Since the same subjects were involved in the entire study, the post-test scale items comprised of closed and open-ended Likert-type questions on a scale of 1 to 5. Samples of the pretest and post-test questionnaires used are provided as Additional files 1, 2, 3, 4 and 5.

To take care of participants with low literacy level, two research assistants were recruited from the local community to guide the respondents through the questionnaires in local languages.

To validate the data collection instruments, we used composite reliability, and Cronbach’s alpha (α) to test internal consistency. We also analyzed validity of the structural model using convergence and discriminant validity. Given our relatively small sample, we performed these tests using PLS algorithm in SmartPLS [36]. Table 2 gives a summary of composite reliability, and Cronbach’s α values generated from the pretest (T_0_), and post-test (T_1_ and T_2_) datasets.

**Table 2:** Reliability test using composite, and Cronbach’s alpha

Predictor variable	Composite reliability			Cronbach alpha		
	T_0_	T_1_	T_2_	T_0_	T_1_	T_2_
Attitude	0.85	0.91	0.92	0.73	0.86	0.88
Efficacy	0.84	0.88	0.92	0.77	0.80	0.88
Sensory	0.85	0.89	0.81	0.75	0.81	*0.67*
Relation	0.88	0.85	0.86	0.80	0.73	0.75
Privacy	0.90	0.86	0.86	0.83	0.76	0.76
Synch	0.85	0.84	0.91	*0.66*	*0.62*	0.80
Represent	0.83	0.86	0.89	0.71	0.76	0.82
Reach	0.82	0.88	0.86	*0.68*	0.80	0.75
Monitor	0.92	0.91	0.91	0.82	0.79	0.80
Fit	0.94	0.93	0.93	0.88	0.84	0.85
Utilization	0.88	0.90	0.94	0.74	0.79	0.86

Source: Primary Data. [NB: The italicized values under Cronbach's alpha falls below the recommended threshhold of 0.70]

The table shows that composite reliability for all the variables were above the recommended 0.70. However, the four values highlighted in Cronbach’s alpha column were slightly less than 0.70. Despite these minor variations, the results indicate good internal consistency of the pretest and post-test scale items.

The results also indicated that Average Variable Extracted (AVE) for all the constructs were above 0.50. According to Chin and Newstead [38], proof of convergent and discriminant validity requires the AVE score for each construct to be above 0.50 (50%). Analysis from the three datasets indicates that each of the eleven constructs has an AVE score above 0.50; hence indicating that TIPFit model has good convergence, and discriminant validity. This confidence in the reliability and validity of the structure of the model was a greenlight to path analysis and hypothesis testing.

#### Data analysis

To analyze the pretest and post-test datasets collected during the experiment, incomplete and wrongly filled questionnaires were eliminated. The valid responses were coded into numerical values and keyed into Statistical Package for Social Scientists (SPSS) to determine the frequency, percentage, and statistical mean of each demographic item.

Regarding predictive modeling, the responses were entered into Microsoft Excel spreadsheet and exported into SmartPLS workspace for analysis using PLS [36–38]. In addition to path analysis, we used Repeated Measures Analysis of Variance (RM-ANOVA), and Bonferroni post hoc test to draw reliable conclusions from the study.

## Results

### Basic demographic characteristics

Most adoption studies have shown that demographic attributes such as gender, age and education influence one’s belief, attitude and ability to perform tasks using technology [9, 14, 15, 17, 21, 22]. In this study, we analyzed these attributes to gain insight on characteristics of the subjects that influence acceptance and use of the planned mHealth intervention. From 79 participants who participated in the pretest conducted before the intervention, we obtained 73 valid questionnaires. The six questionnaires that were disregarded were either incomplete or wrongly filled. Analysis of age distribution using SPSS showed that majority of the respondents were aged between 20 and 25. Table 3 shows the age distribution of 73 valid responses; demonstrating that most of the subjects were within the reproductive age between 20 and 35 years.

**Table 3:** Distribution of participants by age categories

Age category		Frequency	Percent (%)	Valid Percent
Valid	15–19	8	11.0	11.0
	20–25	39	53.4	53.4
	26–30	16	21.9	21.9
	30–35	10	13.7	13.7
	Total	73	100.0	100.0

Source: Primary data

**Fig. 10:**
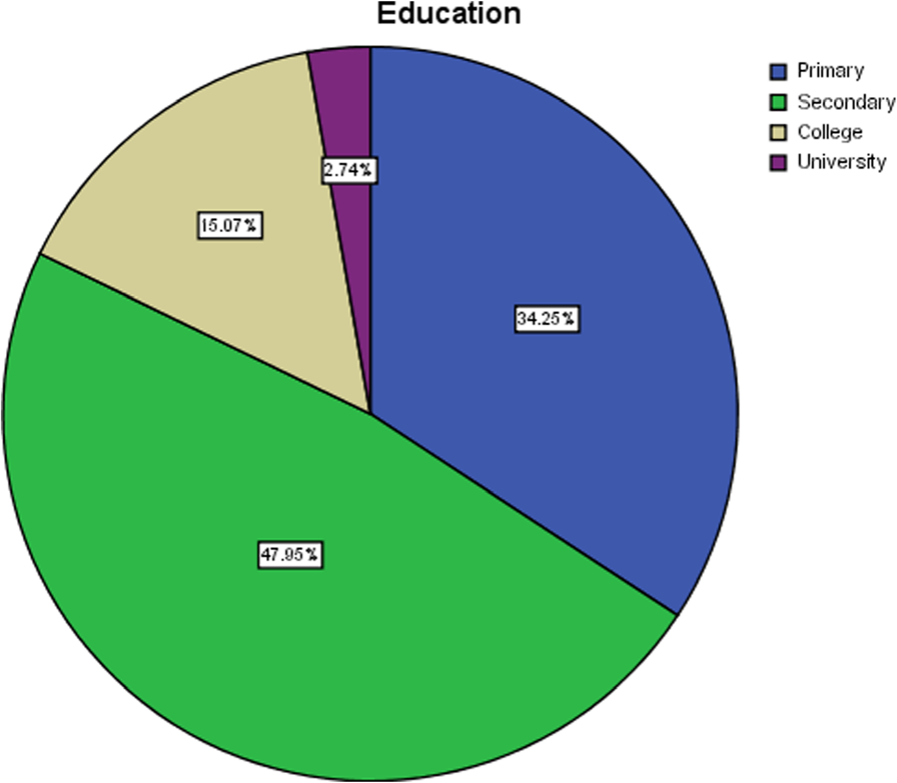
Distribution of participants by education level

Analysis on education revealed that 34.2% of the subjects have studied up to primary school level (Grade 8), and 47.9% up to secondary (Grade 12) as shown in Fig. 10. The pie chart also indicates that 15.1% have studied up to college while only 2.7% have studied up to university. This is a clear reflection that majority of the subjects have low literacy skills that could have been a barrier to effective use of mobile and point-of-care technologies [14–16].

#### Path analysis

**Fig. 11:**
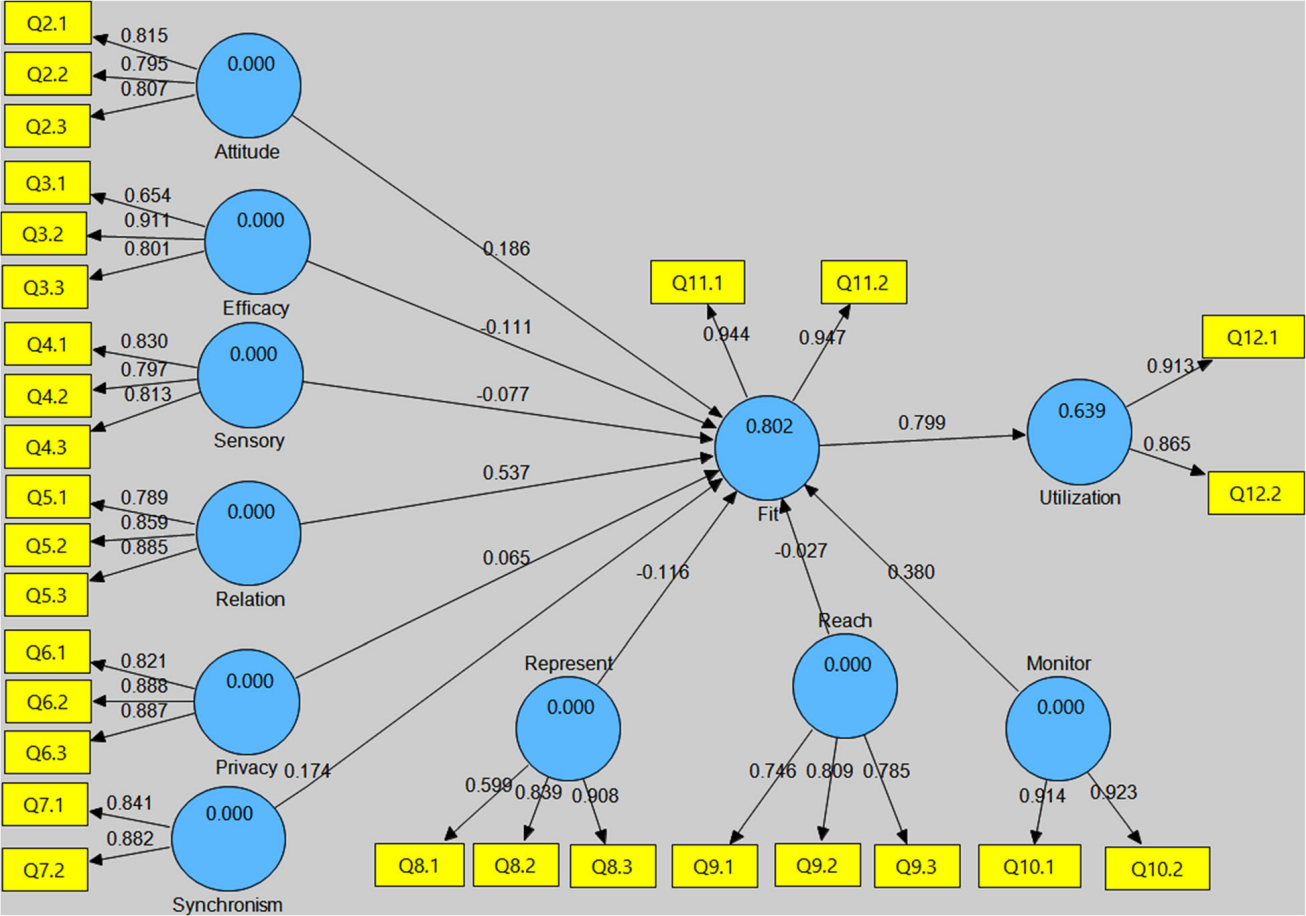
Pretest (prediction) model generated from pretest dataset showing coefficient of determination (R^2^) and path weights. The yellow boxes represent reflective indicators (manifest variables)

To determine the ability of TIPFit in predicting fit and utilization, we used SmartPLS to analyze path weights of the structural model. This is because PLS is variance-based structural equation models that does not impose restrictions on sample size and normality of distribution [37, 38]. Figure 11 shows the structural model generated from the pretest dataset using scale items as reflective indicators of their corresponding predictor variables. The path weights represent coefficients β_1_ to β_10_ in the equation model, and P_1_ to P_10_ on TIPFit model.

The coefficient of determination (R^2^) values of 0.802 and 0.639 indicate that the pretest model has high predictive power of 80.2% on fit, and 63.9% likelihood of utilization. This assumption is based on Overby and Konsynski [22] assertion that a structural model with R^2^ > 0.25 is considered to have good predictive power.

**Fig. 12:**
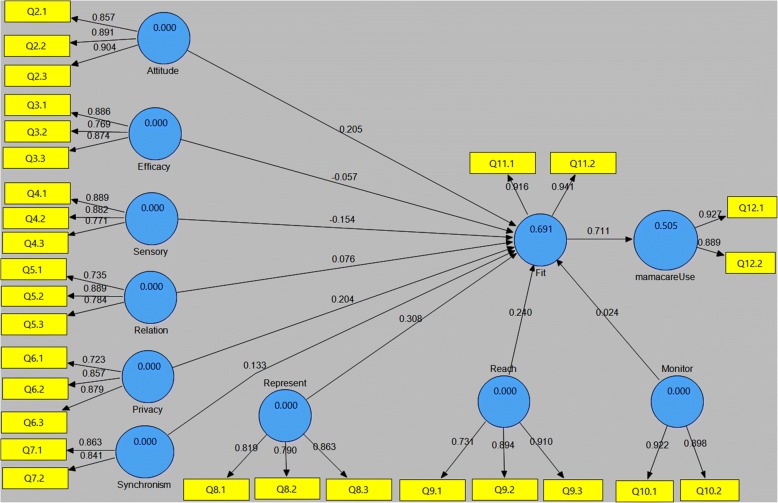
Post-test model generated from initial post-test evaluation showing the coefficients of determination and path weights

After the subjects were exposed to an intervention, dataset collected during the first post-test was cleaned and analyzed using SmartPLS. Figure 12 shows the path weights; R^2^ of 69.1% on fit, and 50.5% of actual utilization. The observed variations between the pretest predictions and actual outcome necessitated improvement of mamacare to address issues raised by the users during the first post-test evaluation.

Three months later, we conducted the second post-test as a follow-up measure. However, due to voluntary exit of six subjects, 73 out of 79 initial participants filled the questionnaires. The post-test2 questionnaire was similar to that used in post-test1 but with additional questions for measuring user satisfaction from enhanced mamacare. The enhancements were mostly on the user interface, language used to send messages, and SMS module for receiving vital signs such as blood pressure, temperature, haemoglobin and blood sugar. The vital signs were used by caregivers to monitor health trends using dynamic charts. This made it easier for caregivers in MNH to easily detect pregnancy and postpartum complications that require urgent attention.

**Fig. 13:**
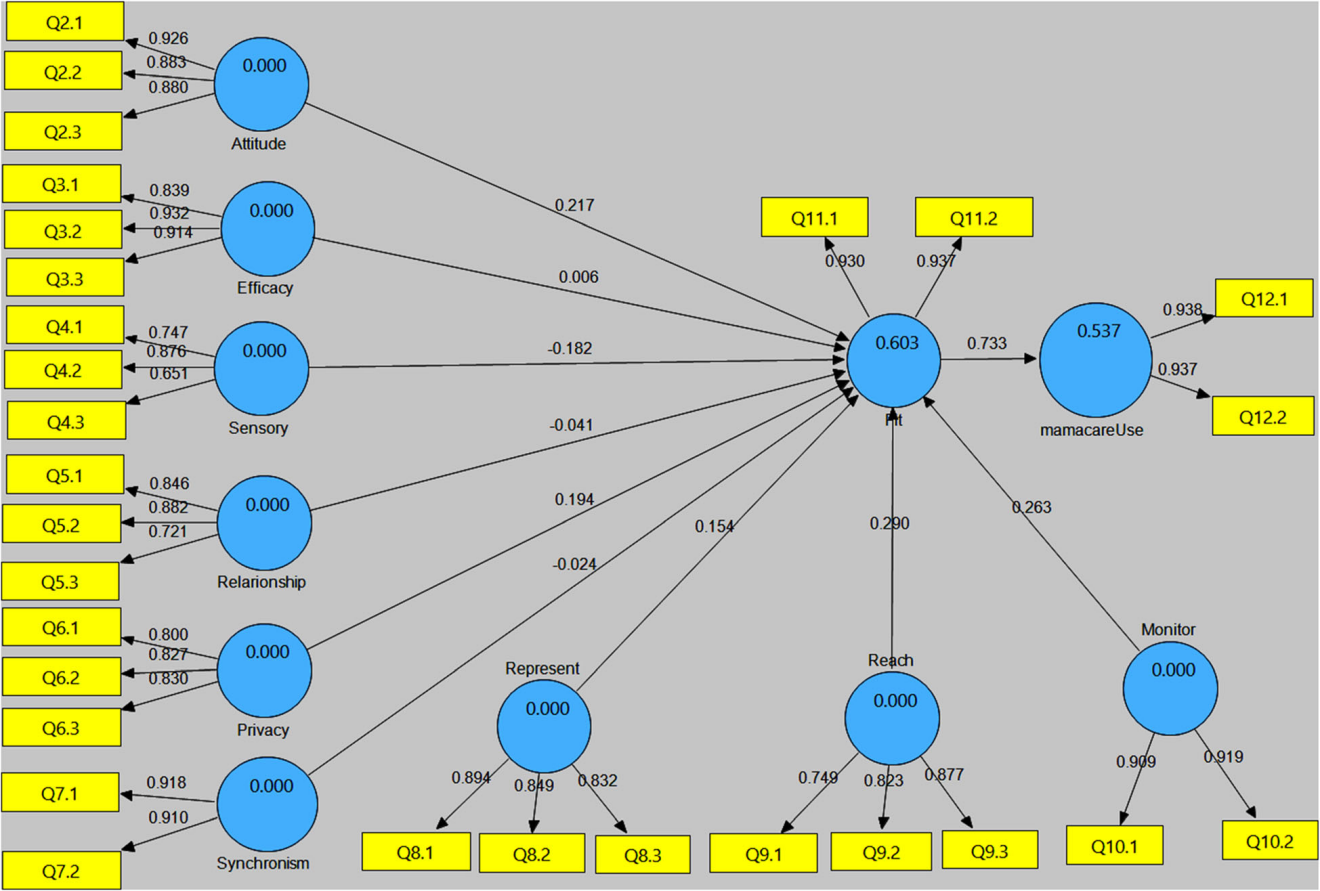
Post-test model generated from the second post-test showing R^2^ and path weights. The model indicates a marginal improvement on utilization of mamacare

Figure 13 shows the model path weights and coefficients of determination after modeling post-test2 dataset using SmartPLS. The results indicate marginal drop on fit from 69.1% recorded in the first post-test to 60.3%. Conversely, the results revealed slight improvement on utilization of mamacare from 50.5% recorded in the first post-test to 53.7%.

In summary, Table 4 shows structural model path weights generated from the pretest and two post-test datasets.

**Table 4:** Summary of path weights from pretest and post-tests models

Test	Atti.	Self	Sense	Rela	Priv	Syn	Rep	Reach	Mo	Fit.
T_0_	0.19	−0.11	−0.08	0.54	0.07	0.17	0.12	−0.03	0.38	*0.79*
T_1_	0.21	−0.06	−0.15	0.08	0.20	0.13	0.31	0.24	0.02	*0.71*
T_2_	0.22	0.01	−0.18	− 0.04	0.19	− 0.02	0.15	0.29	0.26	*0.73*

Source: Primary Data. [NB. The italicized entries in the Fit column indicates that the values are cummulative path weights from the 9 predictor variables X_1_ to X_9_]

The table shows that attitude towards fit was positive before and after intervention. However, Self-efficacy was initially negative but marginally improved after prolonged use of mamacare. We also observe that path weights obtained from sensory requirements were consistently negative before and after the intervention. The cumulative path weights between fit and mHealth utilization shows high positive scores; indicating that fit has strong influence on utilization before, and after intervention.

### Comparative analysis

Due to some inconsistencies observed from the structural path models, we used alternative methods in order to draw reliable conclusions. First, we ran bootstrapping algorithm available in SmartPLS to determine significance of path weights. Table 5 gives a summary of *t* values after bootstrapping the three path models at 5% significance level.

**Table 5:** Significance test results for the bootstrapped path weights

Test	Att	Self	Sens	Rel	Prv	Sync	Rep	Reac	Mon	Fit
T_0_	2.38	−1.71	−1.79	5.07	0.75	2.28	1.43	−0.37	2.73	20.48
T_1_	2.96	−0.83	−3.53	1.09	3.40	1.30	3.91	5.09	0.30	19.52
T_2_	3.16	0.08	−3.65	−0.59	2.14	− 0.25	1.94	3.64	3.86	20.39

Source: Primary Data

Physical inspection on each column indicates temporal changes in hypothesized causation. For example, attitude was consistently positive and significant because its *t* values were greater than the critical value of 1.96 (*t > 1.96)*. Sensory requirements variable consistently returned negative outcomes.

These observations may be interpreted to mean that attitude towards mobile use in maternal care was positive but may not sufficiently address sensory requirements. However, due to inconsistences observed in synchronism, representation and monitoring, we opted to use parametric tests as an alternative to structural path modeling.

### Bonferroni post hoc test

To analyze changes in usage behaviour before and after intervention, we used Bonferroni post hoc test available in SPSS. This test is suitable in studies that seek to establish effect of experimental treatment. Table 6 shows summary of pairwise comparison between the pretest and post-test1 (T_0_-T_1_); post-test1 and post-test2 (T_1_-T_2_); and pretest and post-test2 (T_0_-T_2_).

**Table 6:** Comparison of sample means using Bonferroni post-hoc test

Predictor Variable	Pretest		Post-test1		Post-test2		Mean differences (*p*-value)		
	x̄ =μ	SE	x̄=μ	SE	x̄=μ	SE	T_0_ - T_1_	T_1_-T_2_	T_0_ –T_2_
Attitude	1.56	0.06	1.39	0.06	1.54	0.06	0.11	0.17	0.99
Efficacy	1.55	0.07	1.35	0.06	1.55	0.06	0.10	0.04	1.00
Sensory	2.23	0.12	1.98	0.12	2.11	0.08	0.05	0.21	0.39
Relation	1.81	0.09	1.53	0.06	1.66	0.06	0.11	0.17	0.99
ID. & Privacy	1.89	0.09	1.51	0.07	1.58	0.06	0.01	0.86	0.01
Synchronism	1.84	0.10	1.43	0.07	1.61	0.06	0.00	0.09	0.09
Representation	1.63	0.07	1.47	0.06	1.52	0.05	0.11	0.83	0.48
Reach	1.74	0.07	1.59	0.08	1.65	0.06	0.47	0.93	0.64
Monitor	1.66	0.08	2.00	0.00	1.60	0.06	0.00	0.00	0.92
Fit	1.77	0.09	1.36	0.06	1.54	0.06	0.00	0.06	0.06
mHealth Use	1.69	0.08	1.43	0.07	1.51	0.06	0.02	0.74	0.18

Source: Primary Data

The table shows that there is significant differences between the pretest and post-test1 in sensory requirements, identification and privacy, synchronism, monitoring, fit and utilization of mHealth. However, comparison between T_1_ and T_2_ shows significant differences in *self-efficacy*, and *monitoring* variables. These findings suggest that reactions before the intervention had better predictions after stable use of mamacare. We therefore assume that after improvement of mamacare, usage behaviour almost matched pretest predictions on utilization of mamacare. To investigate these variations, we further analyzed the three datasets using Repeated Measures ANOVA (RM-ANOVA).

### Repeated measures ANOVA

Three essential requirements for using RM-ANOVA are inspection of underlying data for normality of distribution, outliers and sphericity. Although the results from these tests showed the three datasets satisfied the first two requirements, there were some violations of sphericity. Table 7 shows a summary of RM-ANOVA statistics after correcting violations of sphericity in six variables that have *p* values less than 0.05.

**Table 7:** Test of overall treatment effect using RM-ANOVA

	Sphericity		RM-ANOVA:		Effect	Remarks
Predictor	χ2	p-value	F ratio	p-value	Eta^2^	*p* < 0.05
Attitude	0.534	0.766	2.595	0.078	0.036	Not sign.
Self-Efficacy	3.432	0.180	3.258	0.041	0.045	Significant
Sensory	5.109	0.078	1.233	0.295	0.018	Not sign.
Relationship	22.076	< 0.001	4.038	0.029	0.055	GG: Sign.
ID and Privacy	9.980	0.007	7.462	0.001	0.098	GG: Sign.
Synchronism	13.683	0.001	8.022	0.001	0.104	GG: Sign.
Representation	10.664	0.005	2.373	0.105	0.033	GG: Not Sign
Reach	5.034	0.081	1.117	0.330	0.016	Not sign.
Monitoring	20.082	< 0.001	13.384	< 0.001	0.162	GG: Sign.
Fit	8.516	0.014*	10.144	< 0.001	0.128	GG: Sign.
mHealth use	1.350	0.509	4.152	0.018	0.057	Sign.

Source: Primary Data

Visual inspection on RM-ANOVA column indicates that there is no significant differences in four variables with p values less than 0.05. These are attitude, sensory requirements, representation, and reach. This inference implies that mamacare intervention did not change participants’ perception on these predictor variables. In summary, Table 8 shows conclusions drawn from Repeated Measures ANOVA results to either support or reject hypothesized relationships.

**Table 8:** Conclusions drawn from RM-ANOVA analysis

Predictor	H_0_	Prediction hypotheses	Conclusion drawn (p < 0.05)
Attitude	H_0_1	Attitude has no significant change on fit before, and after use of mHealth intervention	Nonsignificant - accept
Self-Efficacy	H_0_2	Self-efficacy has no significant change on fit before, and after use of mHealth intervention	Significant - reject
Sensory	H_0_3	Sensory requirements have no significant change on fit before, and after use of mHealth intervention.	Nonsignificant - accept
Relationship	H_0_4	Relationship requirement has no significant change on fit before, and after use of mHealth intervention	Significant - reject
Identification and Privacy	H_0_5	Identification and privacy has no significant change on fit before, and after use of mHealth intervention	Significant - reject
Synchronism	H_0_6	Synchronism requirement has no significant change on fit before, and after use of mHealth intervention	Significant - reject
Representation	H_0_7	Representation capability of technology has no significant effect on fit before, and after use of mHealth intervention	Nonsignificant - accept
Reach	H_0_8	Reach capability of mHealth technology has no significant change on fit before, and after use of mHealth intervention	Nonsignificant - accept
Monitoring	H_0_9	Monitoring capability of technology has no significant change on fit before, and after use of mHealth intervention	Significant - reject
Fit for Use	H_0_10	Perceived fit has no significant change before, and after use of mHealth intervention	Significant - reject

Source: Primary data

From these inferences, we conclude that *attitude*, *sensory requirements*, *representation* and *reach* variables estimated actual outcome observed after exposing the study cohort to mamacare intervention.

By comparing these results with those drawn from structural path models, we observe similarities and some inconsistences. Despite these variations, conclusions drawn from both structural modeling and parametric analyses demonstrate that TIPFit model is capable of predicting utilization of mHealth interventions in the early design stage.

## Discussions

This study used repeated measures quasi-experiment on a single group to measure the power of TIPFit model in predicting utilization of mHealth interventions. To validate the model, a pretest was administered on a study cohort of 79 subjects before exposure to mamacare intervention. The intervention involved sending and receiving SMS alerts and reminders on maternal care services through mobile phones.

### Predictive power of TIPFit model

The study findings revealed interesting trends before and after exposing the study subjects to mamacare intervention. Inferences on the pretest and post-test structural path models revealed that user’s perception on fit constantly dropped after exposing the subjects to the intervention. Moreover, results from RM-ANOVA revealed the intervention had significant change on seven predictor variables. These are self-efficacy, relationship, identification and privacy, synchronism, monitoring, fit and mamacare utilization.

These results are a confirmation to Davis and Venkatesh assertion that; evaluating user acceptance during design can be used to predict post-implementation acceptance and use of a new system [40]. Furthermore, the study shows some similarities to the findings by Bhattacherjee and Premkumar [15] in their study on predicting usage from belief and attitude. Therefore, the findings from this study confirms that predictive approach to user acceptance testing at the design stage can be used to estimate post-deployment utilization [11, 14, 15].

### Strengths of the study

One of the strengths of this study is emphasis on use of open source software to implement mamacare that runs on low-end mobile devices. Mamacare back-end was implemented using Apache web server, MySQL database, PHP, and SMS Tools3 gateway. To make the front-end adaptive to multiple device profiles, we used Twitter bootstrap; a framework that supports HTML5, CSS3 and JavaScript. This makes mamacare a low-cost digital health solution for supporting maternal and newborn care in low-resource settings.

Another strength of the study is the predictive approach used to develop and evaluate mamacare prototype. This approach is a unique contribution to requirements engineering and user-centred system development methodology. The study also demonstrates how to apply structural equation modeling to predict utilization based on the understanding of user’s behaviour, healthcare processes, and technology contexts.

### Study limitations

Theoretical models focusing on **fit** do not give sufficient attention to the fact that system artifacts must be utilized before they deliver performance impacts [19]. Moreover, there is no evidence that quality of an mHealth system leads to increased voluntary utilization. In our pre-study [13], we observed poor systems being utilized extensively in low-income settings due to donor funding, social benefits, ignorance, and availability. For this reason, we argue that increased utilization of mHealth innovations in low-resource settings may not necessarily result to improved quality of health outcomes. This is because there are other socioeconomic and technical factors that influence delivery of healthcare services such as the cost of care, infrastructure, governance, culture, and skilled workforce. Unfortunately, TIPFit model does not consider these factors but only focuses on the three elements of people, process and technology used to evaluate success of information systems.

Another limitation of this study was on the design used to predict utilization. Although single-group repeated measures design used is closer to randomized experiments, the datasets collected from the same subjects may have had likelihood of reporting bias. To maximize on internal and external validity, it is important to observe caution in sample selection, and time difference allowed before taking measurements. This explains the reason why this study lasted for six months. Some of the shortcomings of longitudinal studies are high cost, and decrease in number of subjects due to natural attrition or voluntary withdrawal.

## Conclusions

This study concludes that there is a myriad of mHealth projects that have failed to inspire utilization due to poor alignment to user needs, clinical tasks, technology and environmental context. To address this gap, we demonstrated that measuring intended users’ perceptions on a planned intervention is crucial to predicting acceptance and use.

In practice, it is crucial for developers of mHealth systems to ensure that user-centred evaluation is performed thoroughly in the early design stage. This is because perceived benefits and user expectations measured during the design stage could provide valuable insights on post-deployment utilization of the intervention [40].

In terms of policy, this study demonstrates that increased utilization of mHealth innovations has the potential to accelerate attainment of Universal Health Coverage (UHC) and Sustainable Development Goals (SDGs) in developing countries. However, success of mHealth interventions depends on how value is driven by aligning the artifacts to health needs and expectations at the design stage.

## Additional files

Additional file 1: Pretest questionnaire used prior to implementation of mamacare to measure perception on usefulness mobile and point-of-care devices in maternal care. (DOC 149 kb)

Additional file 2: Post-test questionnaire used after deployment of mamacare prototype to measure user acceptance, satisfaction and actual utilization. (DOC 150 kb)

Additional file 3: Pretest dataset obtained from randomly selected antenatal and post-natal women. The dataset was used as the baseline for predicting post-deployment utilization of mamacare services. (CSV 5 kb)

Additional file 4: Initial post-test dataset obtained from the study cohort after exposing the subjects to mamacare intervention. (CSV 5 kb)

Additional file 5: Second post-test dataset obtained from the cohort after prolonged exposure to enhanced mamacare services. (CSV 4 kb)

## Abbreviations

ANOVA: Analysis of Variance
eHealth: electronic health
ICT: Information and Communication Technology
ITU: International Telecommunication Union
PLS: Partial Least Squares
SDGs: Sustainable Development Goals
SMS: Short Message Service
TIPFit: Technology, Individual, Process Fit
WHO: World Health Organization
