# Supplementary material for: A model for predicting utilization of mHealth interventions in low-resource settings: case of maternal and newborn care in Kenya
Source: BMC Med Inform Decis Mak. 2018 Jul 17;18:67. doi: 10.1186/s12911-018-0649-z (PMC6050709; doi:10.1186/s12911-018-0649-z)
Supplement: Supplementary file 1 — Pretest questionnaire used prior to implementation of mamacare to measure perception on usefulness mobile and point-of-care devices in maternal care. (DOC 149 kb) [file 12911_2018_649_MOESM1_ESM.doc]

CLIENTS’ OPINION ON USE OF MOBILE PHONES IN

*Questionnaire A*

ANTENATAL AND POSTNATAL CARE

TIME____________ DATE__________________

*| First Page* 🡪

| **INSTRUCTIONS** | | | | |
| --- | --- | --- | --- | --- |
| 1. *This Form consists of 12* ***Parts****. Kindly answer All or ask for help if the question is not clear* 2. *Please DO NOT write your Name* 3. *For Questions based on 5 Options, Please Select ONLY ONE OPTION if you* ***Strongly Agree****,* ***Agree****,* ***Not Sure, Disagree*** or ***Strongly Disagree*** | | | | |
| 1. **Personal Details (This is for analysis only)** | | | | |
| 1.1. **Age (Year**s)  15 – 19  20 – 25  26 – 30  31 – 35  Above 35 | 1.2. **Education**  Primary  Secondary  College  University  None Above | 1.3. **Pregnancies**  1  2  3  4  Above 4 | 1.4. **Children**  0  1  2  3  4 and Above | 1.5. **Own a Mobile?**  Yes  No |
| 1. **Personal Opinion** | | | | |
| *Please give us your opinion on use of mobile phones in Maternal and Child Healthcare (MCH)*  2.1 In my opinion, use of mobile phone services in antenatal and postnatal care can be useful to me  Strongly Agree  Not Sure  Strongly Disagree  Agree  Disagree  2.2 I believe use of mobile phone can make MCH improve service delivery to clients and patients  Strongly Agree  Agree  Not Sure  Disagree  Strongly Disagree  2.3 Use of mobile phone services in maternal care can help me adhere to clinic follow-up schedule  Strongly Agree  Agree  Disagree  Strongly Disagree  Not Sure | | | | |
| 1. **Mobile Phone Use** | | | | |
| *This section aims at getting information on your literacy and ability to use mobile phones*  3.1 Because of my level of education, I easily learn how to use a mobile phone  Strongly Agree  Not Sure  Strongly Disagree  Agree  Disagree  3.2 I have necessary skills in using a mobile phone to read, write and send **sms** messages  Strongly Agree  Agree  Not Sure  Disagree  Strongly Disagree  3.3 I’m comfortable using a mobile phone to access services e.g. mobile money transfer (m-Pesa®)  Strongly Agree  Agree  Not Sure  Disagree  Strongly Disagree | | | | |
| 1. **Follow-up Clinics** | | | | |
| *Please give us your views on whether use of mobile phone can minimize need of going to hospital*  4.1 I believe good use of mobile phone can minimize need of going to hospital to see a doctor/nurse  Strongly Agree  Not Sure  Strongly Disagree  Agree  Disagree  4.2 I would be interested in checking my body condition e.g. BP and send observations to hospital  via mobile phone for advice on any danger signs  Strongly Agree  Not Sure  Agree  Strongly Disagree  Disagree  4.3 Getting preventive care advice via mobile phone is better than going to ask from a doctor/nurse  Strongly Agree  Agree  Disagree  Not Sure  Strongly Disagree | | | | |
| 1. **Relationship with Medical Staff** | | | | |
| *Please indicate your opinion on use of mobile phone in enhancing relationship with medical staff*   - 1. I believe use of mobile phone services can improve my relationship with medical staff in MCH   Strongly Agree  Agree  Not Sure  Disagree  Strongly Disagree   - 1. In case of health-related issues, I have no problem medical staff contacting me via mobile phone   Strongly Agree  Agree  Not Sure  Disagree  Strongly Disagree   - 1. I expect use of mobile phone to help in updating doctor/nurse on my own/baby’s health status   Strongly Agree  Agree  Not Sure  Disagree  Strongly Disagree | | | | |
| 1. **Personal Privacy** | | | | |
| *To what extent do you agree with the following statements regarding privacy of your health status?*   - 1. I easily distinguish between genuine **sms**/**calls** and those received from unknown sources   Strongly Agree  Agree  Not Sure  Disagree  Strongly Disagree   - 1. If my health record is to be stored in a computer, it should be available only to people I trust   Strongly Agree  Agree  Not Sure  Disagree  Strongly Disagree   - 1. I would like to be consulted if MCH section decides to be sending clinic **sms** messages to me   Strongly Agree  Agree  Not Sure  Disagree  Strongly Disagree | | | | |
| 1. **Timeliness** | | | | |
| *To what extent do you agree with the following regarding timeliness of received* ***sms*** *reminders*?   1. I would like to be receiving clinic reminders via **sms** in good time so that I prepare for the visit   Strongly Agree  Strongly Agree  Agree  Not Sure  Disagree  Strongly Disagree   1. I would appreciate getting timely information e.g. danger signs and vaccination via my phone   Agree  Not Sure  Disagree  Strongly Disagree | | | | |
| 1. **Clinic SMS Messages** | | | | |
| *If you would like to be receiving* ***sms*** *reminders, indicate your preference in regard to its content*  8.1 If I’m to receive clinic **sms** reminders, the messages should be in my preferred language  Disagree  Strongly Disagree  Strongly Agree  Not Sure  Agree  8.2. The content of **sms** messages addressed to me should be short and easy to understand  Strongly Agree  Agree    Disagree  Not Sure  Strongly Disagree  8.3. I would like to get clinic **sms** messages that are relevant to my clinic follow-up programme  Strongly Agree  Agree  Not Sure  Strongly Disagree  Disagree | | | | |

*Next Page* 🡪

| 1. **Maternal Care Access** |
| --- |
| *Please help us know how mobile phone can improve access to antenatal and postnatal care*  9.1 If MCH decides to provide maternal care via phone, the service should always be available  Strongly Agree  Agree  Not Sure  Disagree  Strongly Disagree  9.2 I believe use of mobile phone services in MCH can improve access to antenatal/postnatal care  Agree  Strongly Agree  Not Sure  Disagree  Strongly Disagree  9.3 MCH should make it possible to access vital clinic follow-up information on mobile phone  Strongly Agree  Not Sure  Disagree  Strongly Disagree  Agree |
| 1. **Health Monitoring** |
| *Please indicate extent to which mobile phones can help in monitoring and you or your child health*    10.1 I believe adequate use of mobile phone can help in preparation for safe delivery/motherhood  Strongly Agree  Agree  Not Sure  Disagree  Strongly Disagree  10.2 Maternal care services via mobile phone should help in monitoring my own/baby’s progress  Strongly Agree  Agree  Not Sure  Disagree  Strongly Disagree |
| 1. **Expectations** |
| *Overall, indicate extent to which use of mobile services can meet your needs and expectations*  11.1 I hope use of mobile phones to provide maternal care services will be suitable to my needs  Strongly Agree  Agree  Not Sure  Disagree  Strongly Disagree  11.2 I believe use of mobile phone services in antenatal/postnatal care will meet my expectations  Strongly Agree  Agree  Not Sure  Disagree  Strongly Disagree |
| 1. **General Remarks** |
| *Generally, comment on how we can make use of available technologies to improve maternal care*  12.1 Generally, I like the idea of using mobile phones to provide better maternal care services  Agree  Strongly Agree    Not Sure  Disagree  Strongly Disagree  12.2 If provided by MCH, I would like to be getting information and services via mobile phone  Strongly Agree  Not Sure  Strongly Disagree  Agree  Disagree  12.3 *If you agree with (12.2) above, please write the services you would LIKE to be provided*  *through your mobile phone (see sample services below)*  _____________________________________________________________________________  _____________________________________________________________________________  _____________________________________________________________________________  *(Safe delivery, danger signs, medication adherence, vaccination, lab test results, nutrition, preventive care, family planning, education on HIV/Aids etc)* |

🡨 *Last Page |*
