# Supplementary material for: A model for predicting utilization of mHealth interventions in low-resource settings: case of maternal and newborn care in Kenya
Source: BMC Med Inform Decis Mak. 2018 Jul 17;18:67. doi: 10.1186/s12911-018-0649-z (PMC6050709; doi:10.1186/s12911-018-0649-z)
Supplement: Supplementary file 2 — Post-test questionnaire used after deployment of mamacare prototype to measure user acceptance, satisfaction and actual utilization. (DOC 150 kb) [file 12911_2018_649_MOESM2_ESM.doc]

SUITABILITY OF MOBILE PHONES USE

*Questionnaire B*

IN ANTENATAL AND POSTNATAL CARE

TIME____________ DATE__________________

*| First Page* 🡪

| **INSTRUCTIONS** | | | |
| --- | --- | --- | --- |
| 1. *This Form consists of 12* ***Parts****. Kindly answer All or ask for help if the question is not clear* 2. *Please DO NOT write your Name* 3. *For Questions based on 5 Options, Please Select ONLY ONE OPTION if you* ***Strongly Agree****,* ***Agree****,* ***Not Sure, Disagree*** or ***Strongly Disagree*** | | | |
| 1. **Personal Details** (This is for analysis only) | | | |
| 1.1. Age (Years)  15 – 19  20 – 25  26 – 30  31 – 35  Above 35 | 1.2. Education Level  Primary  Secondary  College  University  None Above | 1.3. No. of Pregnancies  1  2  3  4  Above 4 | 1.4. No. of Children  0  1  2  3  4 and Above |
| 1. **Personal Opinion** | | | |
| *Please give us your opinion on use of mobile phones in Maternal and Child Healthcare (MCH)*  2.1 Based on my experience, use of mobile phone services in antenatal and postnatal care is useful  Strongly Agree  Not Sure  Strongly Disagree  Agree  Disagree  2.2 Use of mobile phone is a good idea for improving service delivery in maternal healthcare  Strongly Agree  Agree  Not Sure  Disagree  Strongly Disagree  2.3 Clinic **sms** reminders I receive from MCH has helped me adhere to clinic follow-up schedule  Strongly Agree  Agree  Disagree  Strongly Disagree  Not Sure | | | |
| 1. **Ease of Using Mobile Phone** | | | |
| *This section aims at getting information on your literacy and ability to use mobile phones*  3.1 Because of my level of education, I easily learned how to use my mobile phone  Strongly Agree  Not Sure  Strongly Disagree  Agree  Disagree  3.2 I have necessary skills in using a mobile phone to read, write and send **sms** messages  Strongly Agree  Agree  Not Sure  Disagree  Strongly Disagree  3.3 I’m comfortable using a mobile phone to read **sms** messages sent to me from MCH section  Strongly Agree  Agree  Not Sure  Disagree  Strongly Disagree | | | |
| 1. **Follow-up Clinics** | | | |
| *Please give us your views on whether use of mobile phone minimizes need of going to hospital*  4.1 I believe good use of mobile phone minimizes need of going to hospital to see a doctor/nurse  Strongly Agree  Not Sure  Strongly Disagree  Agree  Disagree  4.2 I’m interested in checking my body condition e.g. BP and send observations via mobile phone to  Strongly Agree  Not Sure  Agree  Strongly Disagree  hospital for advice on any danger signs  Disagree  4.3 Getting preventive care advice via mobile phone is better than going to ask from a doctor/nurse  Strongly Agree  Agree  Disagree  Not Sure  Strongly Disagree | | | |
| 1. **Relationship with Medical Staff** | | | |
| *Please indicate your opinion on use of mobile phone in enhancing relationship with medical staff*   - 1. I believe use of mobile phone has improved my relationship with medical staff in MCH section   Strongly Agree  Agree  Not Sure  Disagree  Strongly Disagree   - 1. In case of health-related issues, a medical staff can easily contact me through mobile phone   Strongly Agree  Agree  Not Sure  Disagree  Strongly Disagree   - 1. I believe use of mobile phone helps in updating doctor/nurse on my own/baby’s health progress   Strongly Agree  Agree  Not Sure  Disagree  Strongly Disagree | | | |
| 1. **Personal Privacy** | | | |
| *To what extent do you agree with the following statements regarding identification and privacy?*   - 1. I easily identify **sms**/**calls** received from MCH section and those from unknown sources   Strongly Agree  Agree  Not Sure  Disagree  Strongly Disagree   - 1. I would like MCH to handle my records stored in the computer as private and confidential   Strongly Agree  Agree  Not Sure  Disagree  Strongly Disagree   - 1. I’m comfortable receiving **sms** reminders from MCH section because I was informed about it   Strongly Agree  Agree  Not Sure  Disagree  Strongly Disagree | | | |
| 1. **Timeliness** | | | |
| *To what extent do you agree with the following regarding timeliness of received* ***sms*** *messages*?   1. I receive clinic **sms** reminder in time which helps me remember to prepare for scheduled visit   Strongly Agree  Strongly Agree  Agree  Not Sure  Disagree  Strongly Disagree   1. I appreciate getting timely information e.g. danger signs and preventive care via mobile phone   Agree  Not Sure  Disagree  Strongly Disagree | | | |
| 1. **Clinic SMS Messages** | | | |
| *Please indicate your opinion on content and format of* ***sms*** *messages received from MCH section*  8.1 Clinic **sms** messages I receive addresses me by name, with content that is easy to understand  Disagree  Strongly Disagree  Strongly Agree  Not Sure  Agree  8.2. I like the idea of choosing my preffered language for receiving clinic **sms** messages from MCH  Strongly Agree  Agree    Disagree  Not Sure  Strongly Disagree  8.3. Clinic **sms** messages I receive are in line with my current antenatal/postnatal clinic follow-up  Strongly Agree  Agree  Not Sure  Strongly Disagree  Disagree | | | |

*Next Page* 🡪

| 1. **Maternal Care Access** |
| --- |
| *Please help us know how mobile phone can improve access to maternal care services*  9.1 Since I was registered for mobile phone services, I always get/got clinic visit messages via **sms**  Strongly Agree  Agree  Not Sure  Disagree  Strongly Disagree    9.2 Use of mobile phone services in MCH section provides better access to antenatal/postnatal care  Agree  Strongly Agree  Not Sure  Disagree  Strongly Disagree  9.3 MCH makes it possible to get antenatal and postnatal care information through mobile phone  Strongly Agree  Not Sure  Disagree  Strongly Disagree  Agree |
| 1. **Health Monitoring** |
| *Please indicate extent to which mobile phone helps in monitoring your own/child health status*    10.1 I believe adequate and timely **calls**/**sms** helps in preparation for safe delivery/motherhood  Strongly Agree  Agree  Not Sure  Disagree  Strongly Disagree  10.2 I believe good use of mobile phone services helps in monitoring of my own/baby’s progress  Strongly Agree  Agree  Not Sure  Disagree  Strongly Disagree |
| 1. **Suitability** |
| *Overall, indicate whether sms messages you have been receiving from MCH section are*  *suitable*  11.1 Based on my experience, clinic **sms** reminders I receive from MCH section are important  Strongly Agree  Agree  Not Sure  Disagree  Strongly Disagree  11.2 I would recommend use of mobile phone services be offered to all antenatal/postnatal clients  Strongly Agree  Agree  Not Sure  Disagree  Strongly Disagree |
| 1. **General Remarks** |
| *Generally, indicate whether you are satisfied with current mobile phone services provided by MCH*  12.1 Generally, I’m satisfied with the current clinic reminders sent via **sms** to me by MCH section  Agree  Strongly Agree    Not Sure  Disagree  Strongly Disagree  12.2 So long as I’m in antenatal/postnatal care, I would like to be getting services via mobile phone  Strongly Agree  Not Sure  Strongly Disagree  Agree  Disagree  12.3 *If you agree with (12.2) above, please list maternal care services you would LIKE to be*  *provided through your mobile phone*. (*For ease of listing, see sample services below)*  _____________________________________________________________________________  _____________________________________________________________________________  _____________________________________________________________________________  *(Safe delivery, danger signs, medication adherence, vaccination, lab test results, nutrition, preventive care, family planning, education on HIV/Aids etc)* |

🡨 *Last Page |*
